# Supplementary material for: The reaction of hydroxyl and methylperoxy radicals is not a major source of atmospheric methanol
Source: Nat Commun. 2018 Oct 19;9:4343. doi: 10.1038/s41467-018-06716-x (PMC6195545; doi:10.1038/s41467-018-06716-x)
Supplement: Supplementary file 1 — Supplementary Information [file 41467_2018_6716_MOESM1_ESM.pdf]

Supplementary Information

The reaction of hydroxyl and methylperoxy radicals is not a major source of atmospheric methanol

Caravan *et al.*

Supplementary Note 1: Determining concentrations in the pulsed photolysis MPIMS experiments

To convert the observed ion signal to a concentration, a reference species, for which the concentration is known, alongside the photoionization cross sections of both the species of interest and the reference species must be utilized. Absolute concentrations of reaction products are derived for the low-pressure experiments, and the initial concentrations of H_2O_2 (30 Torr experiments) and D_2O (740 Torr experiments) are determined using photoionization cross sections. The procedure used in this work to obtain these concentrations will be outlined below.

The H_2O_2 concentration in the 30 Torr experiments was determined in separate measurements at a photoionization energy of 11.5 eV, performed back-to-back with the OH + CH_3OO kinetic experiments, in which H_2O_2, He and a commercially prepared standard of alkenes in Ar, were flowed with flow rates and a reactor pressure such that the H_2O_2 concentration was identical with that used in the kinetic experiments. The concentration of propene (for which the photoionization is well characterized)[1] within the reactor and the photoionization cross sections of H_2O_2[2] and propene[1, 3] were used to determine the concentration of H_2O_2 using the equation below (E1).

$$N_x = \left(\left(\frac{S_x/\sigma_x}{S_y/\sigma_y}\right) . \left(\frac{m_y}{m_x}\right)^{\beta(p,T)}\right) . N_y \qquad \text{E1}$$

Where N is the concentration, S is the ion signal, σ is the photoionization cross section at a given photon energy and m is the mass. The subscripts x and y refer to the species of interest and the reference species, in this case H_2O_2 and propene, respectively. β, the mass discrimination factor, which accounts for mass-dependent sampling efficiencies of the instrument, was obtained through separate experiments using a commercially prepared standard of rare gases in He.[3] This factor depends on the expansion characteristics of the sampled gases, and by extension on the bulk composition. Therefore, for concentration determinations in the high-O_2 conditions of the reaction mixture (where calibration with the high photon energies needed for our reference standard is impractical), we choose a reference (H_2O_2) as close in mass as feasible to the target compounds (CH_2O and CH_3OH), so that the factor (m_y/m_x) is nearly unity. See S3 of the Supplemental Material from Rotavera *et al.*[4] for further

details regarding E1. For the 740 Torr experiments, a similar procedure was employed to determine [D_2O] using its absolute photoionization cross section.[5]

Dilute mixtures of methyl iodide (CH_3I, or $^{13}CH_3I$) in He were prepared using a gas manifold and pressures to determine the dilution. The photoionization cross section of methyl iodide has been measured at 118.2 nm by Gans *et al.*,[6] which can be used to place the photoionization spectrum of CH_3I on an absolute scale (Supplementary Figure 1). The measured signal on the minor isotopes of methyl iodide ($^{12}CH_3I$ for the ^{13}C labelled measurements) can then be used in conjunction with the calibrated [H_2O_2] to provide an estimate for the mass discrimination factor in the reacting mixture, and derive concentrations of species such as I_2 to within an estimated factor of 2. The initial concentrations of OH and CH_3 were deduced by examining the depletion profile of the ion signals of CH_3I after photolysis in the non-background subtracted dataset (Supplementary Figure 2), and scaling the H_2O_2 photolytic depletion using the known cross sections.[7] The fit to this depletion profile includes an instrument response function and effects of the photolysis beam divergence through the long tubular reactor.[2] The beam divergence causes an axial gradient in photolyzed fraction, and because the laser propagates along the flow direction, that gradient is mapped into a time-dependent change in photolysis fraction at the sampling location. This effect is negligible in the smaller high-pressure reactor. The two photolysis fraction representations in Supplementary Figure 2 give significantly different predictions for absolute concentrations (Figure 1a) but essentially identical predictions for the ratio of formaldehyde to methanol (Supplementary Figure 3).

An excess of O_2 was used ($[O_2]/[CH_3]_0 > 10^3$-10^4) and it could be assumed that all CH_3 were converted to CH_3OO on a pseudo-instantaneous timescale. The methanol and formaldehyde concentrations were determined from the ion signal through reference to H_2O_2. The relative cross sections of methanol, formaldehyde, and hydrogen peroxide have been measured accurately by Dodson *et al.*,[2] and placed on an absolute scale by reference of the methanol cross section to the cross section of propene. Because of the interrelationship of the cross-section determinations, many of the uncertainties among the concentrations cancel, and the relative 15% uncertainties are smaller than the estimated ~20% uncertainty in the overall scaling to absolute concentration. The signals at 11 eV photon energy were definitively attributed to formaldehyde (m/z = 31, Supplementary Figure 4) and methanol (m/z = 33, Supplementary Figure 5) by comparison of the full photoionization spectrum to known reference spectra.

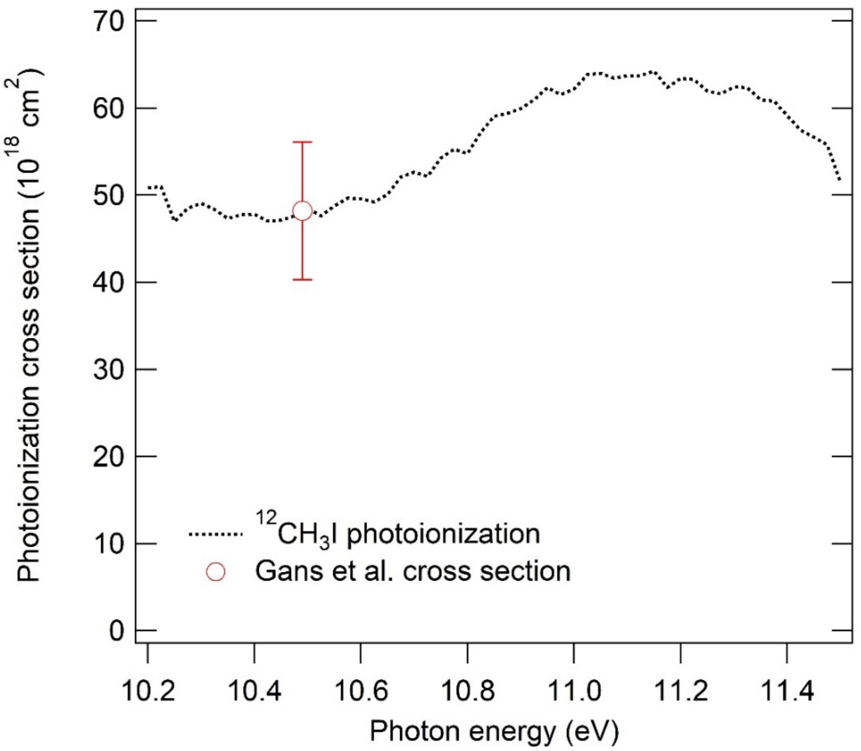

Supplementary Figure 1. Photoionization spectrum of CH_3I. The measured relative photoionization spectrum of CH_3I is placed on an absolute scale by comparison to the determination by Gans *et al.*[6] The error bars are 2σ and are those given by Gans *et al.*[6]

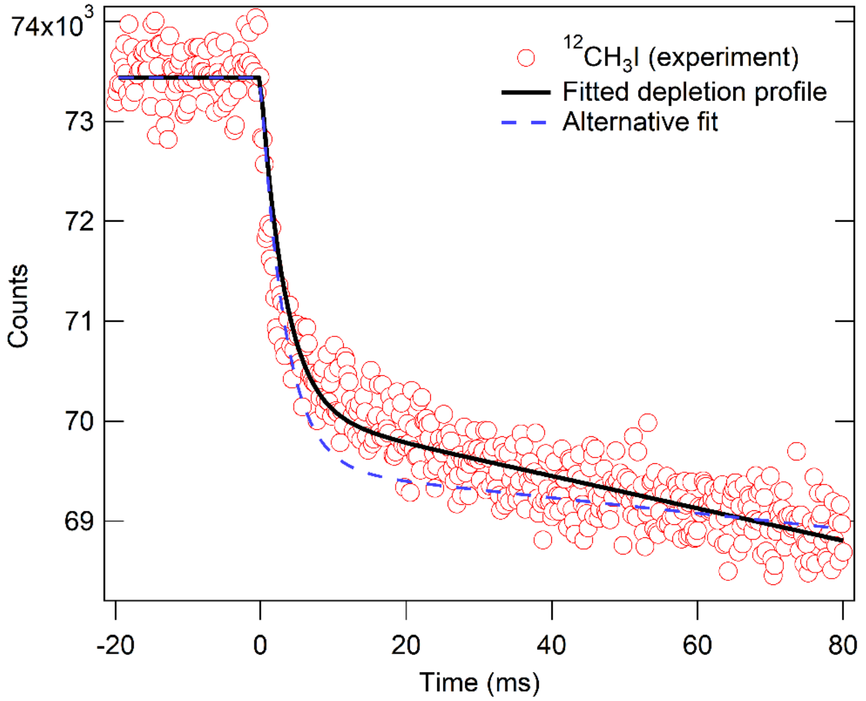

Supplementary Figure 2. Photolytic depletion of CH_3I (summed over all experiments). The data are fitted (solid line) including instrument response function and effects of photolysis beam divergence along the reactor flow axis. The dashed line is an alternative fit constrained to a smaller divergence.

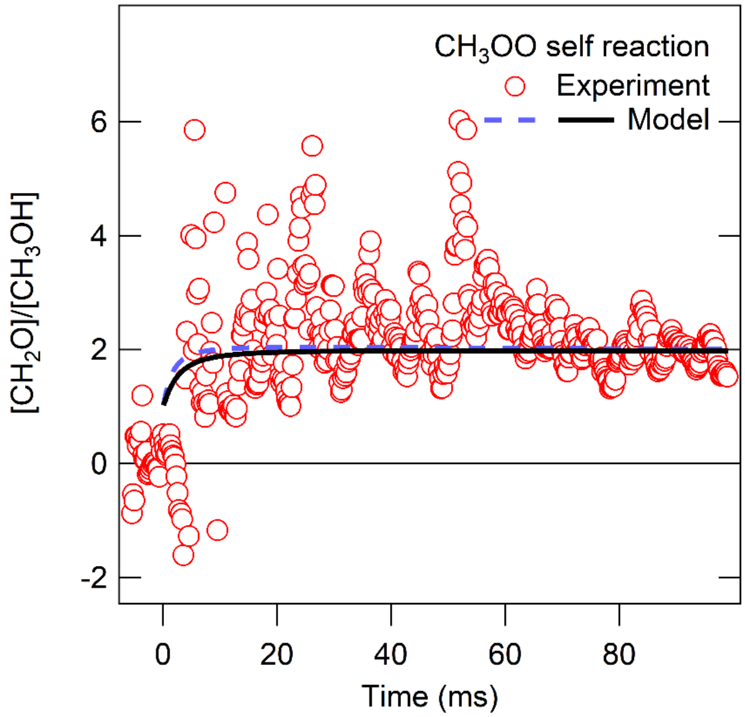

Supplementary Figure 3. Ratio of [CH_2O]/[CH_3OH] from CH_3OO self-reaction. The ratio is simulated using the two fits to the photolysis in Supplementary Figure 2,and is essentially insensitive to changes to photolysis fraction within uncertainty.

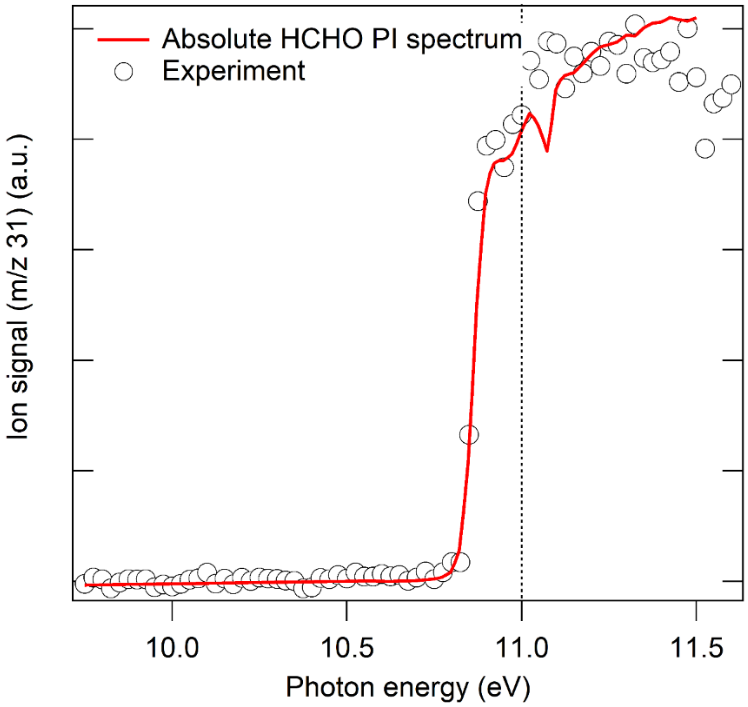

Supplementary Figure 4. Photoionization spectra of formaldehyde and m/z = 31. Comparison of observed signal at m/z = 31 in the reaction of $^{13}CH_3OO$ with OH to a reference spectrum of formaldehyde. Kinetic measurements were acquired at 11 eV photon energy (vertical dashed line).

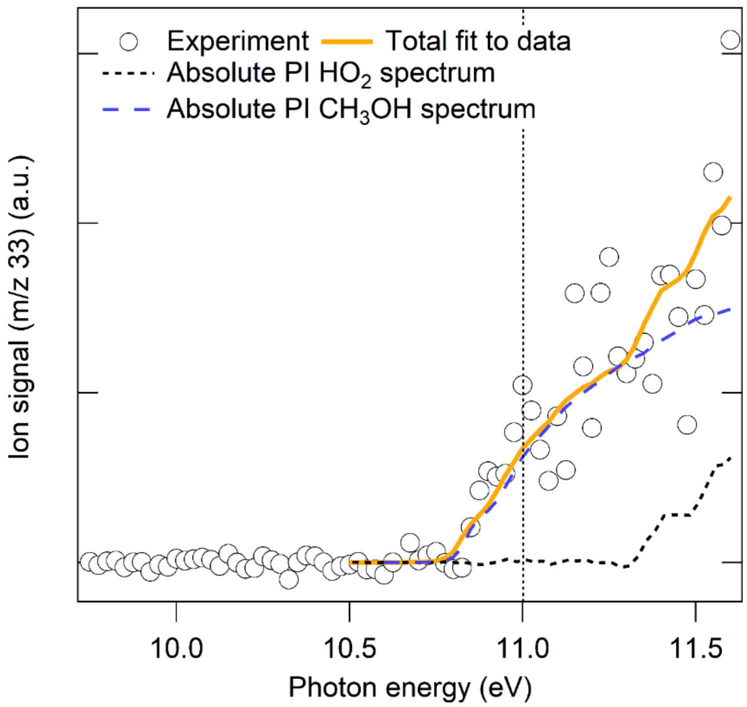

Supplementary Figure 5. Photoionization spectra of HO_2, CH_3OH and m/z =33. Comparison of observed signal at m/z = 33 in the reaction of $^{13}CH_3OO$ with OH to reference spectra of methanol and HO_2. Kinetic measurements were acquired at 11 eV photon energy (vertical dashed line).

Supplementary Note 2: Kinetic models
The continuous photolysis chamber experiments have been modelled using the mechanism in Supplementary Table 1:

Supplementary Table 1. Mechanism used to extract the CH_3OH yield from chamber studies

No.	Reaction	k / cm³ s⁻¹	Ref
1	$O_3 \rightarrow 2\ OH$	$1 – 2 \times 10^{-4}\ s^{-1}$	Adjusted to O_3 decay
2a	$CH_3O_2 + OH \rightarrow HO_2 + CH_3O$	1.3×10^{-10}	Assaf *et al.* [8, 9]
2b	$CH_3O_2 + OH \rightarrow CH_3OH + O_2$	2.5×10^{-11}	Adjusted to CH_3OH profile
3	$OH + O_3 \rightarrow HO_2 + O_2$	7.3×10^{-14}	Atkinson *et al.*[10]
4	$OH + CH_4 + O_2 \rightarrow CH_3O_2 + H_2O$	6.28×10^{-15}	Atkinson *et al.*[11]
5a	$2\ CH_3O_2 \rightarrow 2\ CH_3O + O_2$	1.3×10^{-13}	Atkinson *et al.*[11]
5b	$2\ CH_3O_2 \rightarrow CH_2O + CH_3OH$	2.2×10^{-13}	Atkinson *et al.*[11]
6	$CH_3O + O_2 \rightarrow CH_2O + HO_2$	1.9×10^{-15}	Atkinson *et al.*[11]
7	$CH_3O_2 + HO_2 \rightarrow CH_3OOH + O_2$	5.2×10^{-12}	Atkinson *et al.*[11]
8	$2\ HO_2 \rightarrow H_2O_2 + O_2$	2.552×10^{-12}	Atkinson *et al.*[10]
9	$HO_2 + O_3 \rightarrow OH + 2\ O_2$	2.0×10^{-15}	Atkinson *et al.*[10]
10	$OH + HO_2 \rightarrow H_2O + O_2$	1.0×10^{-10}	Atkinson *et al.*[10]
11	$OH + CH_3OH + O_2 \rightarrow CH_2O + HO_2 + H_2O$	9.3×10^{-13}	Atkinson *et al.*[11]
12	$OH + CH_2O + O_2 \rightarrow H_2O + CO + HO_2$	8.5×10^{-12}	Atkinson *et al.*[11]
13	$OH + CO + O_2 \rightarrow CO_2 + HO_2$	2.41×10^{-13}	Burkholder *et al.*[7]
14a	$CH_3OOH + OH \rightarrow CH_3O_2 + H_2O$	6×10^{-12}	Atkinson *et al.*[10]
14b	$CH_3OOH + OH \rightarrow CH_2O + H_2O + OH$	4×10^{-12}	Atkinson *et al.*[10]

Modelling the laser photolysis experiments was done by employing the relevant reactions in Supplementary Table 1 and some additional reactions specific to the photolytic experiments, as shown in Supplementary Table 2 below.

Supplementary Table 2. Additional kinetic parameters used to model MPIMS pulsed laser photolysis experiments

**Reaction**	**Rate coefficient or branching fraction**	**Reference**
$CH_3OO + CH_3OO \rightarrow$ products	3.5×10^{-13} cm^3 s^{-1}	11
$\rightarrow CH_3OH + HCHO + O_2$	$\phi = 0.56$	12 (a)
$\rightarrow 2\ CH_3O + O_2$	$\phi = 0.38$	12 (a)
$\rightarrow CH_3OOCH_3 + O_2$	$\phi = 0.06$	12 (a)
$CH_3OO + HO_2 \rightarrow$ products	5.5×10^{-12} cm^3 s^{-1}	13
$\rightarrow CH_3OOH + O_2$	$\phi = 0.89$	14
$\rightarrow HCHO + H_2O_2$	$\phi = 0.11$	14
$CH_3 + O_2 \rightarrow CH_3OO$		
30 Torr	1.5×10^{-13} cm^3 s^{-1}	15
740 Torr	8.5×10^{-13} cm^3 s^{-1}	15
$CH_3OO + OH \rightarrow$ products	1.6×10^{-10} cm^3 s^{-1}	9
$\rightarrow CH_3O + HO_2$	$(1-\phi_{CH3OH})$	fitted
$\rightarrow CH_3OH + O_2$	ϕ_{CH3OH}	fitted
$OH + H_2O_2 \rightarrow H_2O + HO_2$	1.7×10^{-12} cm^3 s^{-1}	10
$OH + CH_3I \rightarrow CH_2I + H_2O$	9.9×10^{-14} cm^3 s^{-1}	16
$CH_3O + HO_2$	1.1×10^{-10} cm^3 s^{-1}	17
$CH_3O + CH_3O \rightarrow CH_2O + CH_3OH$	7.0×10^{-11} cm^3 s^{-1}	17
**Iodine reactions**		
$I + CH_3OO \rightarrow CH_3OOI$	8×10^{-12} cm^3 s^{-1}	18 (b)
$CH_3OOI + I \rightarrow I_2 + CH_3OO$	1.5×10^{-10} cm^3 s^{-1}	18
$OH + I_2 \rightarrow HOI + I$	2.1×10^{-10} cm^3 s^{-1}	19
**F atom reactions**		
$F + CH_4 \rightarrow HF + CH_3$	6.7×10^{-11} cm^3 s^{-1}	7
$F + D_2O \rightarrow DF + OD$	3.5×10^{-12} cm^3 s^{-1}	20

[a]The branching fractions differ from those in Supplementary Table 1 by the inclusion of a minor component to form CH_3OOCH_3, which is observed in the photoionization experiments, at the upper limit given in reference [12].
[b]Adjusted for 30 Torr conditions to match observed time profile of I_2.

Supplementary Note 3: Analysis of the continuous photolysis chamber experiments

The chamber was sampled through a 1 m long PFA line (perfluoroalkoxy, 1/8" OD) held at 50°C. The PTR-TOFMS sampling flow rate was set at 150 mL min^{-1}, leading to a residence time less than 1s. The instrument was operated at reactor pressure and temperature of 1.33 mbar and 40°C, respectively, leading to an E/N ratio of 131 Td. This instrument exhibits a resolution (m/Δm) spanning 3000-4000.

The only parameters adjusted are (a) the O_3 photolysis rate such that the O_3 decay is reproduced and (b) the rate coefficient of (R2b) such that the CH_3OH concentration time profile is reproduced. A total of 6 experiments were carried out under different conditions, leading to an average CH_3OH yield of 16.5 ± 3 %. The summary is given below in Supplementary Table 3.

Supplementary Table 3. Summary of conditions at which the continuous photolysis chamber experiments were carried out.

**Experiment**	**Initial concentrations (ppm)**			**Chamber volume (L)**	**Number of lamps on**	**Yield (%)**
	**$[CH_4]_0$**	**$[O_3]_0$**	**$[H_2O]_0$**			
1	15	1.5	1080	320	4	16
2	12	0.89	1240	320	4	18
3	15	3.4	1300	320	4	15
4	15	0.90	1250	320	2	17
5	7.5	0.54	1600	320	2	15
6	7.5	0.21	1130	150	2	18
Average (±2σ)						16.5±3

For several species, the measured signal is significant before turning on the lamps:

a) CH_3OH is present in small concentrations in the CH_4 cylinder and thus injected at the same time as CH_4. The initially concentration present before turning on the lamps (few ppb) is taken into account when modelling the chemistry by entering an initial CH_3OH concentration.

b) CH_2O is also always present in low concentrations in the chamber (same order of magnitude as CH_3OH), plus some additional CH_2O is formed when lamps are turned on, possibly desorbing from walls. This is taken into account by (1) entering a non-zero initial concentration and (2) addition of an artificial source of formation in the mechanism, see below.

c) Peaks corresponding to the chemical formula CH_3O_2 (m/z = 47.01164) and CH_5O_3 (m/z = 65.02234) are clearly present already before turning on the lamps, but their intensity slightly increases after turning on the lamps. This can be seen in Supplementary Figure 6. No clear explanation can be given for the presence of these peaks before turning on the lamps. $CH_3O_2^+$ could be protonated formic acid or a fragment, which could be formed upon protonation of the trioxide with subsequent elimination of H_2O. We find evidence for the latter pathway through *ab initio* calculations as discussed in Supplementary Note 3. However, the major increase in concentration takes place rapidly after turning on the lamps, so it seems to be a species

that desorbs from the chamber wall, instead of something formed on the chemistry time scale. We can conclude that the PTR-TOFMS detects, in the best case, only trace amounts of the trioxide or its possible fragment.

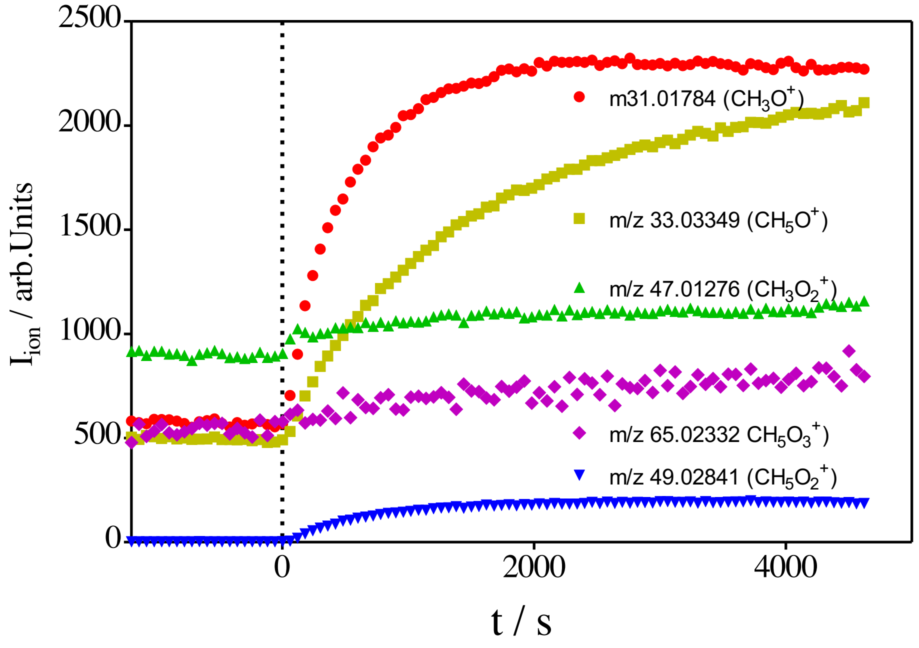

Supplementary Figure 6. Time profiles for five key m/z from the chamber/PTR-TOFMS experiments. The data correspond to the following species: red dots: CH_2O, yellow squares: CH_3OH, blue triangles: CH_3OOH, green triangles: HC(O)OH, magenta diamonds: CH_3OOOH. Scales are in arbitrary units, calibrations of the transmission curve have been carried out daily, including CH_3OH.

The presence of CH_2O and CH_3OH has some impact on the CH_3OH yield because it is a sink for OH radicals, thus leaving fewer OH radicals to react with CH_3O_2. This is taken into account in the model by introducing a non-zero concentration for both species. However, the model always underpredicts CH_2O formation. We always observe a formation of CH_2O when turning on the lamps, possibly due to desorption from the chamber walls. This extra CH_2O has however only a very minor influence on the CH_3OH profile: all OH having reacted with CH_2O (R12) will instantaneously (on our time scale) be recycled by reaction of the co-product HO_2 with O_3 (R9). Supplementary Figure 7 demonstrates this; the dashed lines represent the model from Supplementary Table 1 with $k_{2b} = 2.5 \times 10^{-11}$ cm^3s^{-1}, *i.e.* a CH_3OH yield of 16%. For the full lines, a reaction has been added to the model (A + B → CH_2O) to better reproduce the CH_2O profile. No changes have been made to the rate coefficient k_{2b}. It can be seen that the impact on the CH_3OH profile is very minor.

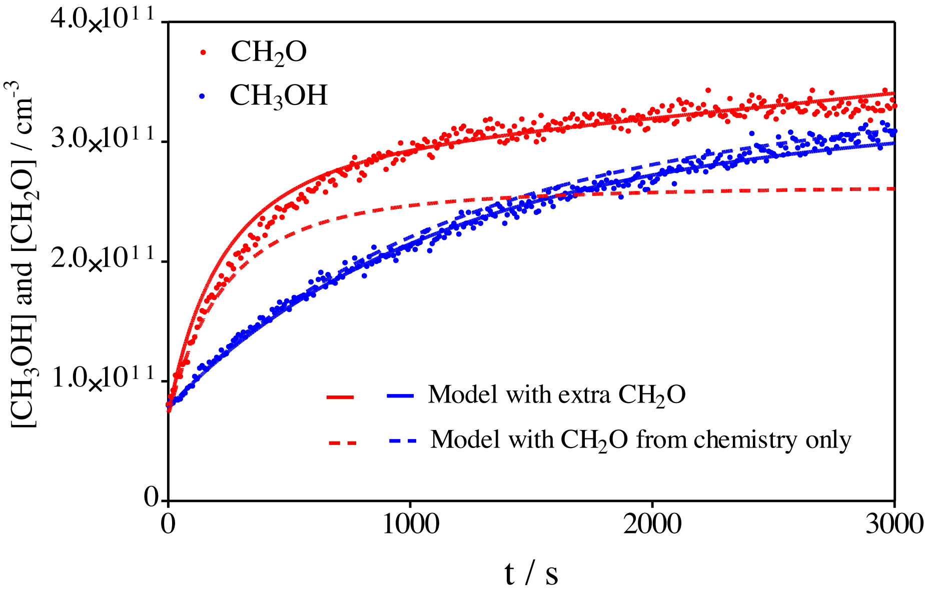

Supplementary Figure 7. **Model from Supplementary Table 1.** Dashed lines with CH_2O from chemistry only, full lines with CH_2O artificially increased to better match the measurements.

Supplementary Note 4: Analysis of pulsed photolysis MPIMS experiments

The CH_2O and CH_3OH concentrations are simulated with a chemical kinetic model and the branching fraction ϕ_{CH3OH} is varied to obtain the best fit. For the 30 Torr data the best estimate of ϕ_{CH3OH} was obtained by computational fit. The uncertainty in the best fit is completely dominated by model uncertainties. The fit was carried out while varying the most sensitive parameters within their literature uncertainties (Supplementary Table 4). The uncertainty in the photoionization cross section ratio[2] overwhelms other sources of uncertainty in the branching fraction. Reactions involving I atoms are also modelled; Supplementary Figure 8 shows the modelled and measured I_2 profile. Although iodine reactions with OH have been implicated as confounding factors in the kinetics of OH with CH_3OO, in the present case I_2 is almost completely removed between laser pulses and the effect of iodine side reactions on OH removal is negligible. Supplementary Figure 9 shows that the decay of OH in the absence of O_2 (and therefore in the absence of CH_3OO) is unchanged by photolysis of CH_3I.

Supplementary Table 4. Best fit branching fractions at 30 Torr for various conditions and parameter values.

$[CH_3I]$	1.3×10^{14} cm^{-3}	9.8×10^{13} cm^{-3}	1.6×10^{14} cm^{-3}	9.8×10^{13} cm^{-3}	6.5×10^{13} cm^{-3}	2.0×10^{14} cm^{-3}	
$[H_2O_2]$	8.3×10^{13} cm^{-3}	8.2×10^{13} cm^{-3}	8.3×10^{13} cm^{-3}	8.3×10^{13} cm^{-3}	7.9×10^{13} cm^{-3}	8.4×10^{13} cm^{-3}	
	ϕ_{CH3OH}	ϕ_{CH3OH}	ϕ_{CH3OH}	ϕ_{CH3OH}	ϕ_{CH3OH}	ϕ_{CH3OH}	**Mean**
**Nominal model**	**0.095**	**0.071**	**0.075**	**0.11**	**0.11**	**0.11**	**0.094**
Vary $\sigma_{HCHO}/ \sigma_{CH3OH}$ ± 15%	0.14	0.12	0.14	0.14	0.15	0.16	0.14
	0.063	0.033	0.042	0.072	0.084	0.07	0.061
Vary ($I + CH_3O$) rate coefficient	0.11	0.081	0.091	0.11	0.11	0.12	0.10
	0.084	0.055	0.061	0.088	0.096	0.089	0.079
Vary ($HO_2 + CH_3O$) rate coefficient	0.085	0.056	0.067	0.09	0.095	0.096	0.081
	0.106	0.083	0.089	0.11	0.12	0.12	0.11
**Uncertainties**							
plus	0.047	0.053	0.071	0.034	0.038	0.058	**0.050**
minus	0.036	0.043	0.036	0.042	0.038	0.041	**0.039**

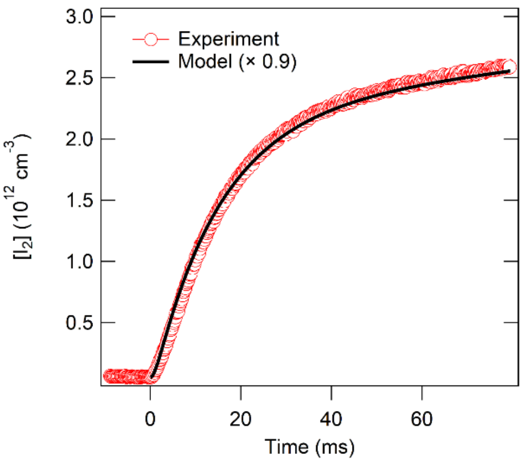

Supplementary Figure 8. Measured I_2 profile compared with a kinetic model. Calibration uncertainty estimated as ~50%.

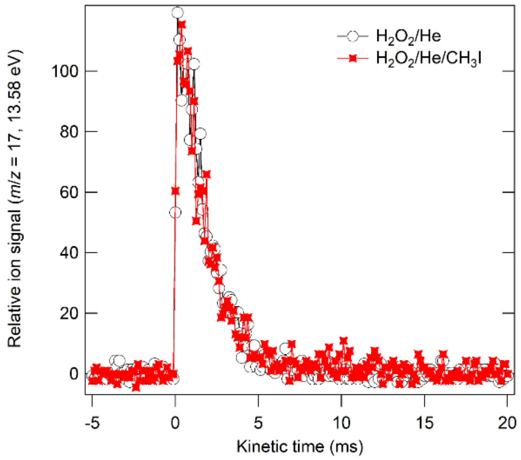

Supplementary Figure 9. Comparison of OH decay with and without CH_3I photolysis.

For the 740 Torr data the model is simpler but the computational fit did not readily converge. The best fit was therefore estimated by eye. Supplementary Figure 10 shows the nominal best fit and the ~1% change in best fits from varying the cross-section ratio within its uncertainty. To have an equal effect on the best fit to the branching fraction, variation of other parameters must be well outside their uncertainty bounds, for example changing [D_2O] or the photolysis fraction from their measured values by 50% (Supplementary Figure 11). Both parameters are known to within 20%.

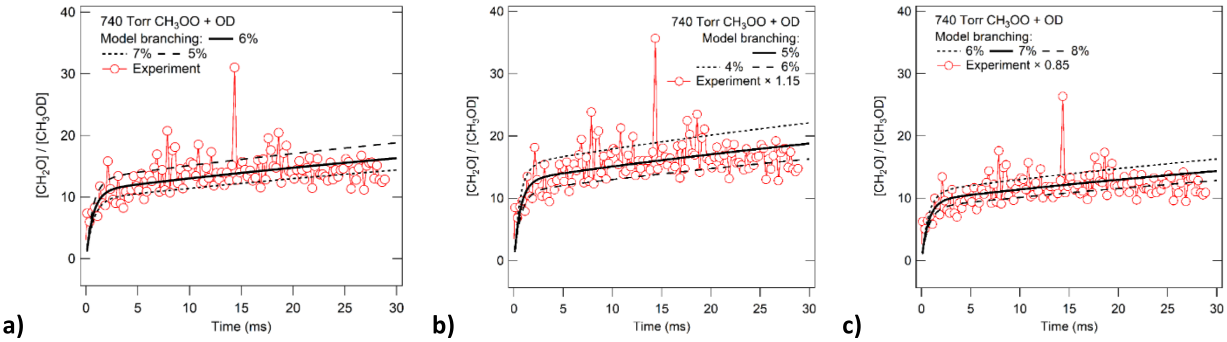

Supplementary Figure 10. Modelled and experimental ratios of CH_2O to CH_3OD in the 740 Torr reaction of CH_3OO with OD. a) nominal parameters, b), c) varying the cross-section ratio $\sigma_{HCHO}/\sigma_{CH3OH}$ within its uncertainty.

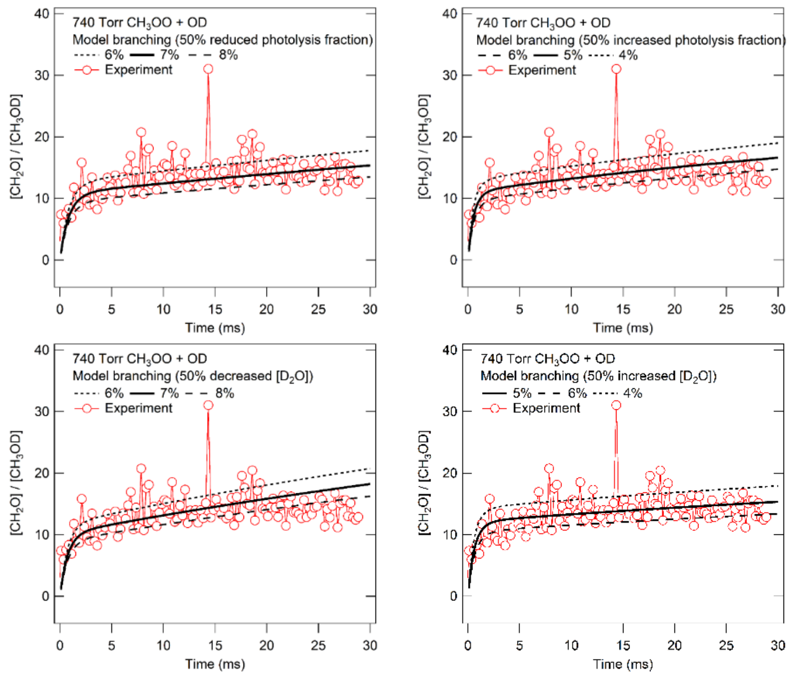

Supplementary Figure 11. Effect of changing modeled photolysis fraction and [D_2O] far outside their uncertainties.

Supplemental Note 5: Calculations of protonated trioxide decomposition pathways

We have undertaken *ab initio* calculations to determine the stability of the protonated trioxide. Three protonation sites have been considered, on each of the oxygen atoms, α, β and ɣ with respect to the methyl group. According to the work of Müller *et al.* ,[21] only the singlet electronic state of the trioxide is pertinent, and so our calculations have only considered the singlet closed shell electronic state of the trioxide.

Geometry optimization and energy calculations have been performed at the M06-2X/6-311++G** level of theory. The likelihood of protonation at each of these sites has been investigated through calculations of the proton affinity of each isomer with respect to water as H_3O^+ is the proton source in the PTR-TOFMS detection chamber experiments. Protonation at the β site is found to be endothermic with respect to water by 9.4 kcal mol^{-1}, whilst protonation at both the α and ɣ sites are determined to be exothermic relative to water. Trioxide which is protonated at the ɣ site is found to yield a van der Waals-like complex between H_2O and $[CH_3O_2]^+$ fragments; protonation at the α site leads to an initially stable species.

As protonation of the trioxide is anticipated to be a barrierless processes, the branching fractions for the protonation sites are likely to be determined by long-range interactions, and so branching fractions for the α and ɣ sites cannot be readily obtained without rigorous dynamics calculations. We can however conclude the following: some of the trioxide formed in the chamber experiments will likely be protonated at the ɣ site, leading to an unstable species which will decompose to water + $CH_3O_2^+$. Some of the trioxide is also anticipated to be protonated at the α site, which will lead to an apparently stable protonated species.

However, due to the role of protonated water in the proton transfer process and, additionally, the presence of appreciable concentrations of H_2O in the chamber experiments (2.5-3.8 x 10^{16} molecule cm^{-3}, and higher in the PTR-TOFMS chamber due to the injection of water to produce H_3O^+) it is necessary to consider the potential role of water reactions with the α-protonated trioxide. We find a highly exothermic process with a submerged barrier (Supplementary Figure 12) for the decomposition of $[CH_3OHOOH...H_2O]^+$ (formed either from the proton transfer process, or through interaction of water with the α-protonated trioxide, in both cases with considerable internal energy, see Supplementary Figure 12), leading to CH_5O^+ + H_2O + O_2.

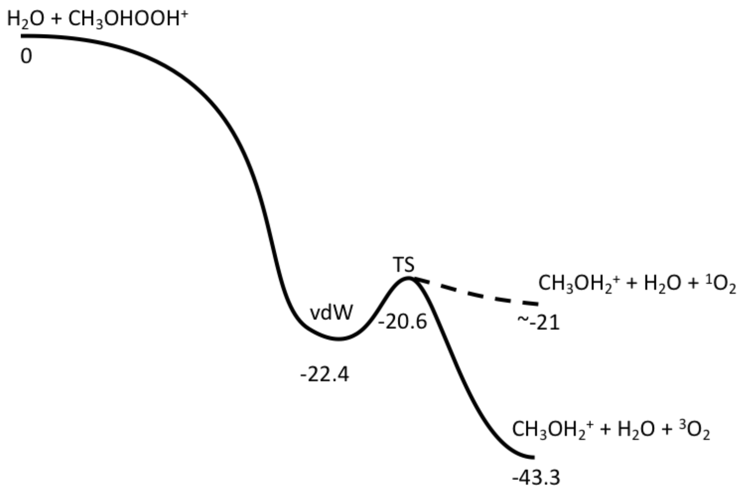

Supplementary Figure 12. Calculated [$CH_3OHOOH...H_2O]^+$ pathway. ZPE-inclusive stationary point energies for the decomposition of $[CH_3OHOOH...H_2O]^+$ obtained at the M06-2x/6-311++G** level of theory.

Trioxide which is not lost via heterogeneous pathways would thus be detected in the PTR system as either $CH_3O_2^+$ (from the ɣ -protonated trioxide) or protonated methanol (from the α -protonated trioxide). This is consistent with our observation of larger apparent methanol yields in the PTR-TOFMS chamber experiments than either the low pressure or ambient pressure MPIMS experiments.

Unfortunately, we cannot determine the branching fraction of trioxide in the OH + CH_3OO reaction in the chamber experiment using this interference due to two factors: (1) the branching between α and ɣ-protonated trioxide is unknown and (2) the detection efficiency of the protonated trioxide fragments is also not known.

Supplementary Note 6: Observation of trioxide in MPIMS experiments

We have carried out further experiments and analysis that show the 7% yield originating from the MPIMS experiments (at 30 and 740 Torr) reflects the direct yield from the reaction of OH and CH_3OO. Experiments have been conducted at 10, 30 and 740 Torr to investigate the formation of trioxide. In the chamber experiments, a very small signal was observed at the mass of the protonated trioxide (65), but our calculations (discussed in Supplementary Note 5) show that it is likely that the majority of any surviving trioxide is converted to $CH_3O_2^+$ and CH_5O^+ following protonation in the PTR-TOFMS system.

MPIMS experiments have been undertaken using three radical precursors; $XeF_2/CH_4/D_2O$ (740 Torr) to generate CH_3OO and OD, $XeF_2/CH_4/H_2O$ (10 Torr) to generate CH_3OO and OH, and $^{13}CH_3I/H_2O_2$ (30 Torr) to generate $^{13}CH_3OO$ and OH. In all experiments, the exact mass of the side-product CH_3OOCH_3 (or the relevant isotopic analogues) originating from the self-reaction of CH_3OO, was utilized to correct the mass axis to determine the exact mass at which the relevant trioxide isotope should appear if formed.

In the low-pressure experiments (10 and 30 Torr), no evidence for trioxide formation is found (Supplementary Figures 13 and 14, below). This is consistent with the branching fractions of 0.002 % and 0.016%, respectively, using the pressure and temperature dependent expression of Müller *et al.*[21] At 740 Torr, where the Müller *et al.* expression predicts a yield of 9.6 % trioxide (without correction for bath gas), a significant peak is detected at the exact m/z corresponding to CH_3OOOD – the isotopologue of trioxide from OD + CH_3OO (Supplementary Figure 15). The signal is time-resolved (Supplementary Figure 16), with an almost instantaneous production following laser photolysis – matching the initial signal of CH_3OD (from OD + CH_3OO), as would be anticipated from the rapid OD + CH_3OO reaction. At longer kinetic times, the CH_3OD signal continues to increase (due to secondary sources from reactions such as DO_2 + CH_3O), whereas the trioxide signal decays, either due to removal reactions or wall loss.

We do not have a reference spectrum to compare the observed signal to that of trioxide, however, we have performed an adiabatic energy calculation of the trioxide at the CBS-QB3 level of theory, to ascertain the photon energy at which the trioxide should be ionized, and thus detected, in the MPIMS experiments. Energy calculations are performed for the neutral and cation of trioxide, in which the geometries are not fixed. The difference in the calculated energies of the cation and neutral corresponds to the adiabatic ionization energy and adiabatic

ionization energy of 9.9 eV is calculated for the trioxide. A stable cationic structure is calculated, indicating that the trioxide can be detected at its parent mass, consistent with our observations of CH_3O_3D at 11.0 eV at 740 Torr. Assuming a comparable photoionization cross section for methanol (which is known) and the trioxide, the yield of trioxide in the 740 Torr experiments will be comparable to that established for methanol (6-7 %) based on their initial signals (Supplementary Figure 16). Error bounds of approximately a factor of 2 are reasonable for the estimated trioxide cross-section, and so trioxide yields in the range 3-12% are feasible. This is consistent with the value of 9.6% predicted by the expression of Müller *et al.*[21], not accounting for any reduced efficiency in collisional stabilisation of the trioxide by He bath gas, as used in the present experiment, relative to air.

Observation of the trioxide at 740 Torr in the MPIMS signal confirms that trioxide is indeed formed from the OH + CH_3OO reaction at higher pressures. Therefore, the trioxide will also be produced in the ambient pressure chamber experiments. As detailed in Supplementary Note 5, we calculate that the protonated trioxide can decompose either to $[CH_3O_2]^+$ or to protonated methanol, depending on the site of protonation. Because of our observation of trioxide in our higher pressure MPIMS experiments, the possibility of heterogeneous conversion of trioxide in the chamber measurements, and our calculations demonstrating that decomposition of the protonated trioxide can lead to artificially high methanol signal in the PTR-TOFMS detection, we conclude that the observed yield of ~6-9% methanol in the MPIMS experiments is a more robust determination of the direct methanol yield from the OH + CH_3OO reaction, and that the 17% yield from the PTR-MS chamber experiments are subject to contributions from the trioxide product.

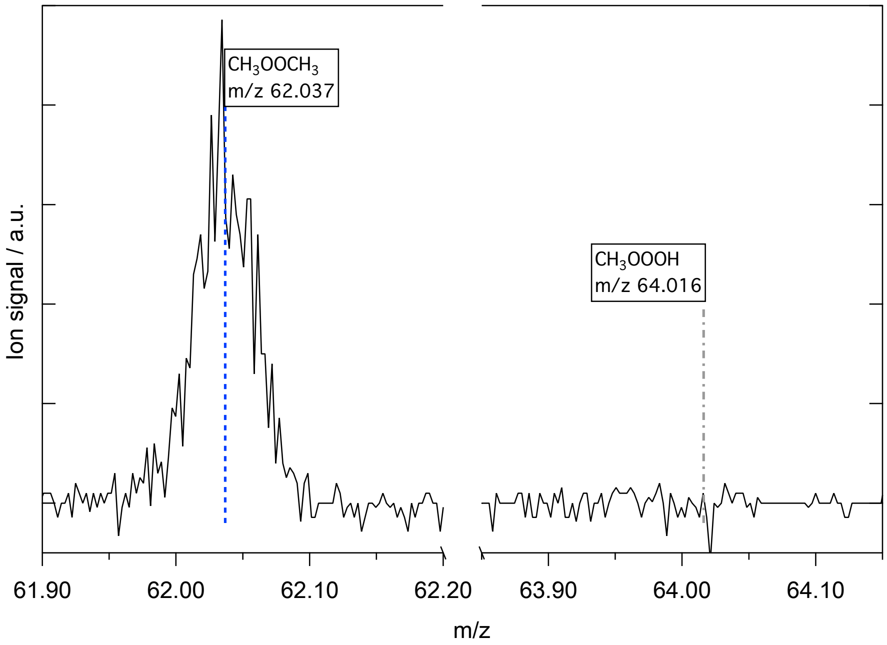

Supplementary Figure 13. Integrated ion signal from the reaction of OH + CH_3OO at 10 Torr, using $XeF_2/CH_4/H_2O$ precursors measured with a photoionization energy of 11.5 eV.

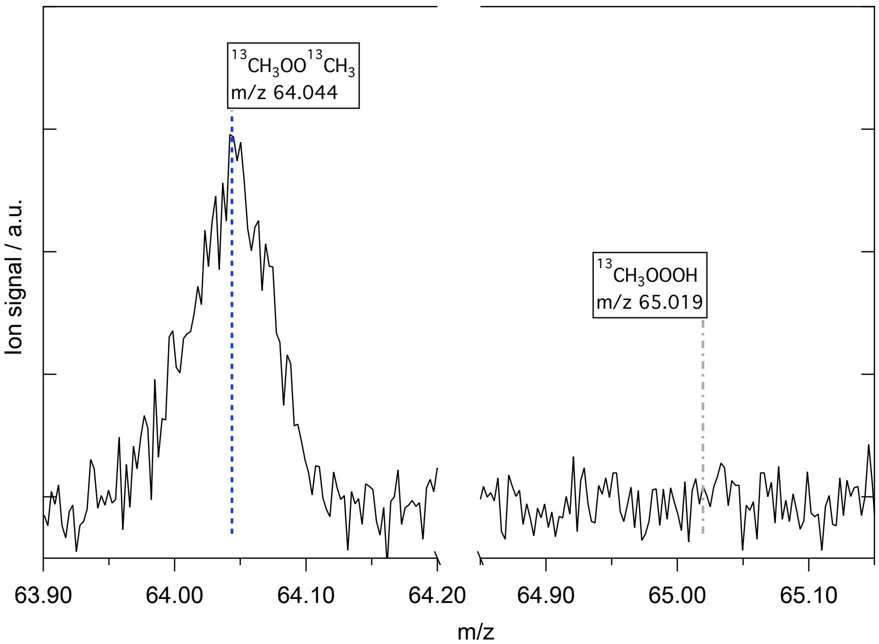

Supplementary Figure 14. Integrated ion signal from the reaction of OH + $^{13}CH_3OO$ at 30 Torr, using $^{13}CH_3I/H_2O_2$ precursors measured with a photoionization energy of 11.0 eV.

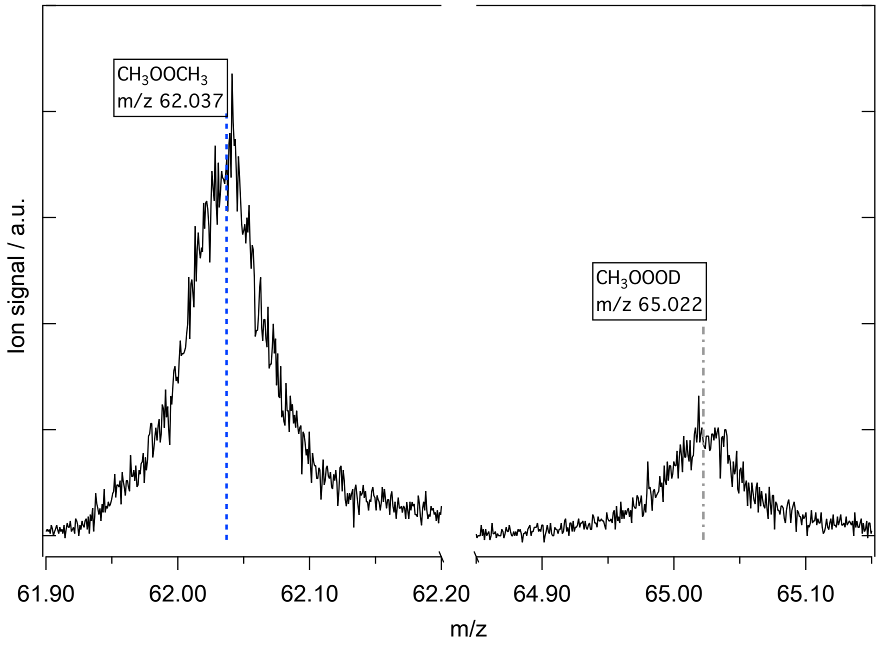

Supplementary Figure 15. Integrated ion signal from the reaction of OD + CH_3OO at 740 Torr, using $XeF_2/CH_4/H_2O$ precursors measured with a photoionization energy of 11.0 eV.

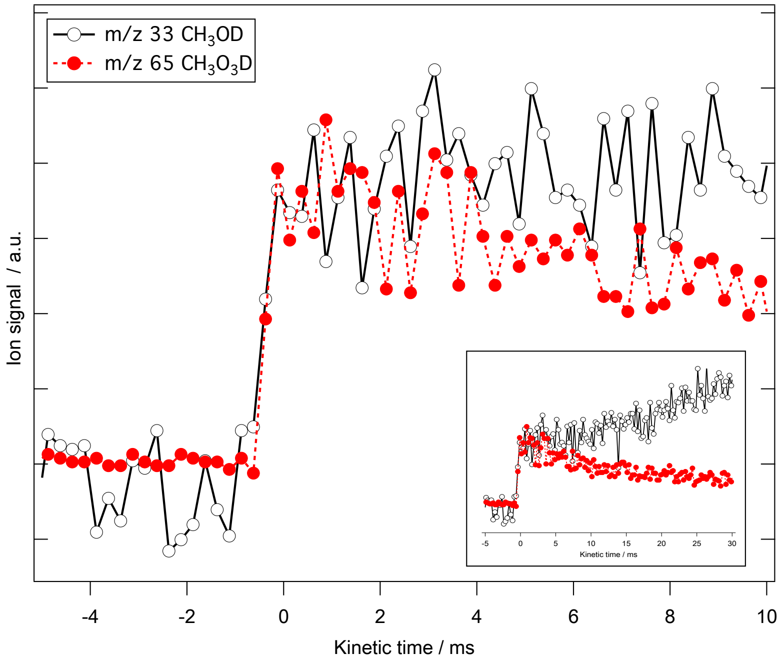

Supplementary Figure 16. **Kinetic profiles of CH_3O_3D and CH_3OD.** Temporal profiles of CH_3O_3D (red closed circles) and CH_3OD (black open circles) from the reaction of OD + CH_3OO at 740 Torr using $XeF_2/CH_4/H_2O$ precursors measured with a photoionization energy of 11.0 eV.

Supplementary Discussion 1: Further discussion of model integration results

For OH at the surface there are reductions of up to 8% in tropical remote oceans due to reaction with CH_3OO, with a slight increment (1%) in the southern hemisphere (SH) oceanic region (Figure 4) where NOx is high in the model. The high levels of NOx are found over the southern hemisphere continents due to biomass burning. The high levels of NOx combined with the large emissions of VOCs (such as isoprene and monoterpenes) in the southern hemisphere continents result in the formation of organic nitrates which decompose away from source regions, releasing NOx in the southern hemispheric oceans. For HO_2 at the surface there is a significant increase (up to 25%) over large areas of the tropical Pacific, Atlantic and Indian Oceans (Figure 4) which are due to the increased production through reaction 2. The decrease in surface ozone levels by up to 4% are observed (Figure 4) in remote tropical oceanic regions that are driven by two major NO_2 formation reactions, CH_3OO + NO and HO_2 + NO (contributing 22% and 56% of the total NO_2 formation flux, respectively) and one significant loss reaction of ozone by HO_2 in the troposphere. The increased concentration of HO_2 due to the addition of reaction (2) increases the flux of the reaction HO_2 + NO by 5%, but the decreased concentration of CH_3OO decreases the flux of the reaction CH_3OO + NO by 16%. The reaction of O_3 with HO_2 accounts for 16% of the total ozone losses in the troposphere, thus the additional source of HO_2 from reaction (2) increases the flux of the reaction HO_2 + O_3 by 4%. These three combined effects lead to reduce the tropospheric ozone on a global scale. A slight increase is observed for CO at southern latitudes with the peak value (2%) at remote tropical oceans (Figure 4). The change in CO is a balance between production via volatile organic compounds' (VOCs) oxidation and removal via reaction with OH. The reaction 2 and the oxidation of methanol (only from reaction 4) produce an additional amount of HCHO (267 Tg/yr for 7% methanol yield, 276 Tg/yr for 17% methanol yield, which is ~13% of the total model HCHO), but the reduced CH_3OO concentration decreases the dominating HCHO production flux of CH_3OO + NO (908 Tg/yr, 44% of the total model HCHO) by 16%. These combined effects resulted in a small change to the surface level of HCHO (Figure 4) ranging from -2.0% up to about 1.5%. For CH_3OOH there are two competing factors, as it is being formed by the reaction between CH_3OO (reduced in the model by inclusion of the OH + CH_3OO reaction, Figure 4) and HO_2 radicals (increased in several regions). The resulting pattern shows reductions of CH_3OOH over oceanic regions (up to 17%, Figure 4). For other organic hydroperoxides (except CH_3OOH) in general the case is much clearer with significant increases (Figure 4) (up to 40%) over the same regions where HO_2 (precursor) is elevated.

The implications of a 17% methanol yield (as observed in the chamber experiments, uncorrected for the trioxide interference) have been considered in the global model and the impact on key species is shown in Supplementary Figure 17.

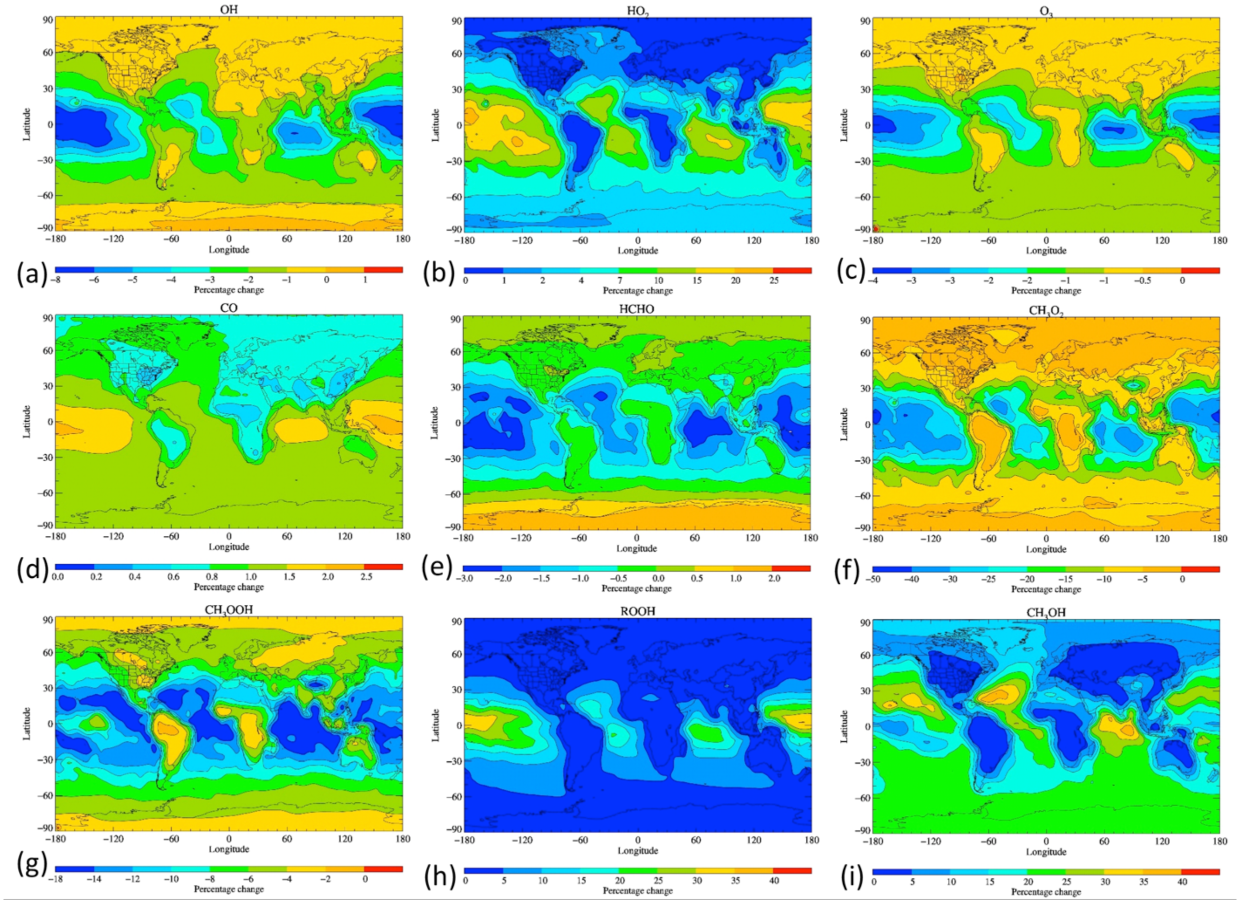

Supplementary Figure 17. The impact of the title reaction on atmospheric key species with a 17% methanol yield. Annual surface percentage changes in (a) OH (b) HO_2 (c) O_3 (d) CO (e) HCHO (f) CH_3O_2 (g) CH_3OOH (h) ROOH (excluding CH_3OOH) and (i) CH_3OH upon inclusion of the OH + CH_3OO reaction with assumed branching fractions ϕ_2 = 0.83, ϕ_3 = 0.00, ϕ_4 = 0.17, ϕ_5 = 0.00.

References

1. Person JC & Nicole PP. Isotope Effects in the Photoionization Yields and the Absorption Cross Sections for Acetylene, Propyne and Propene. *J. Chem. Phys.* **53**, 1767-1774 (1970).

2. Dodson LG*, et al.* VUV Photoionization Cross Sections of HO_2, H_2O_2, and H_2CO. *J. Phys. Chem. A* **119**, 1279-1291 (2015).

3. Welz O*, et al.* Low-Temperature Combustion Chemistry of Biofuels: Pathways in the Initial Low-Temperature (550 K – 750 K) Oxidation Chemistry of Isopentanol. *Phys. Chem. Chem. Phys.* **14**, 3112-3127 (2012).

4. Rotavera B*, et al.* Influence of oxygenation in cyclic hydrocarbons on chain-termination reactions from R + O_2: tetrahydropyran and cyclohexane. *Proc. Combust. Inst.* **36**, 597-606 (2017).

5. Katayama DH, Huffman RE & O'Bryan CL. Absorption and photoionization cross sections for H_2O and D_2O in the vacuum ultraviolet. *J. Chem. Phys.* **59**, 4309-4319 (1973).

6. Gans B*, et al.* Determination of the Absolute Photoionization Cross Sections of CH_3 and I Produced from a Pyrolysis Source, by Combined Synchrotron and Vacuum Ultraviolet Laser Studies. *J. Phys. Chem. A* **114**, 3237-3246 (2010).

7. Burkholder JB*, et al.* Chemical Kinetics and Photochemical Data for Use in Atmospheric Studies, Evaluation No. 18. Jet Propulsion Laboratory, Pasadena. (2015)

8. Assaf E*, et al.* The Reaction between CH_3O_2 and OH Radicals: Product Yields and Atmospheric Implications. *Environ. Sci. Technol.* **51**, 2170-2177 (2017).

9. Assaf E, Song B, Tomas A, Schoemaecker C & Fittschen C. Rate Constant of the Reaction between CH_3O_2 Radicals and OH Radicals Revisited. *J. Phys. Chem. A* **120**, 8923-8932 (2016).

10. Atkinson R*, et al.* Evaluated kinetic and photochemical data for atmospheric chemistry: Volume I – gas phase reactions of Ox, HOx, NOx and SOx species. *Atmos. Chem. Phys.* **4**, 1461-1738 (2004).

11. Atkinson R*, et al.* Evaluated kinetic and photochemical data for atmospheric chemistry: Volume II–gas phase reactions of organic species. *Atmos. Chem. Phys.* **6**, 3625-4055 (2006).

12. Tyndall GS, Wallington TJ & Ball JC. FTIR Product Study of the Reactions CH_3O_2 + CH_3O_2 and CH_3O_2 + O_3. *J. Phys. Chem. A* **102**, 2547-2554 (1998).

13. Raventós-Duran MT, McGillen M, Percival CJ, Hamer PD & Shallcross DE. Kinetics of the CH_3O_2 + HO_2 reaction: A temperature and pressure dependence study using chemical ionization mass spectrometry. *Int. J. Chem. Kinet.* **39**, 571-579 (2007).

14. Elrod MJ, Ranschaert DL & Schneider NJ. Direct kinetics study of the temperature dependence of the CH_2O branching channel for the CH_3O_2 + HO_2 reaction. *Int. J. Chem. Kinet.* **33**, 363-376 (2001).

15. Kaiser EW. Pressure dependence of the rate constants for the reactions methyl + oxygen and methyl + nitric oxide from 3 to 10^4 torr. *J. Chem. Phys.* **97**, 11681-11688 (1993).

16. Cotter ESN, Canosa-Mas CE, Manners CR, Wayne RP & Shallcross DE. Kinetic Study of the Reactions of OH with the Simple Alkyl Iodides: CH_3I, C_2H_5I, 1-C_3H_7I and 2-C_3H_7I. *Atmos. Environ.* **37**, 1125 - 1133 (2003).

17. Assaf E, Schoemaecker C, Vereecken L & Fittschen C. The Reaction of Fluorine Atoms with Methanol: Yield of CH_3O / CH_2OH and Rate Constant of the Reactions $CH_3O + CH_3O$ and $CH_3O + HO_2$ *Phys. Chem. Chem. Phys.*, (2018).

18. Dillon TJ, Tucceri ME & Crowley JN. Laser induced fluorescence studies of iodine oxide chemistry. *Phys. Chem. Chem. Phys.* **8**, 5185 - 5198 (2006).

19. Atkinson R*, et al.* Evaluated kinetic and photochemical data for atmospheric chemistry: Volume III - gas phase reactions of inorganic halogens. *Atmos. Chem. Phys.* **7**, 981 - 1191 (2007).

20. Stevens PS, Brune WH & Anderson JG. Kinetic and mechanistic investigations of $F + H_2O/D_2O$ and $F + H_2/D_2$ over the temperature range 240-373 K. *J. Phys. Chem.* **93**, 4068 - 4079 (1989).

21. Müller J-F, Liu Z, Nguyen VS, Stavrakou T, Harvey JN & Peeters J. The reaction of methyl peroxy and hydroxyl radicals as a major source of atmospheric methanol. *Nature Commun.* **7**, 13213 (2016).
